# Using the Visual World Paradigm to Study Retrieval Interference in Spoken Language Comprehension

**DOI:** 10.3389/fpsyg.2016.00873

**Published:** 2016-06-14

**Authors:** Irina A. Sekerina, Luca Campanelli, Julie A. Van Dyke

**Affiliations:** ^1^Department of Psychology, College of Staten Island, City University of New YorkStaten Island, NY, USA; ^2^Linguistics Program, The Graduate Center, City University of New YorkNY, USA; ^3^Neurolinguistics Laboratory, National Research University Higher School of EconomicsMoscow, Russia; ^4^Speech-Language-Hearing Sciences, The Graduate Center, City University of New YorkNY, USA; ^5^Haskins LaboratoriesNew Haven, CT, USA

**Keywords:** memory retrieval, spoken language comprehension, visual world paradigm, eye-tracking, cleft sentences

## Abstract

The cue-based retrieval theory (Lewis et al., 2006) predicts that interference from similar distractors should create difficulty for argument integration, however this hypothesis has only been examined in the written modality. The current study uses the Visual World Paradigm (VWP) to assess its feasibility to study retrieval interference arising from distractors present in a visual display during spoken language comprehension. The study aims to extend findings from Van Dyke and McElree (2006), which utilized a dual-task paradigm with written sentences in which they manipulated the relationship between extra-sentential distractors and the semantic retrieval cues from a verb, to the spoken modality. Results indicate that retrieval interference effects do occur in the spoken modality, manifesting immediately upon encountering the verbal retrieval cue for inaccurate trials when the distractors are present in the visual field. We also observed indicators of repair processes in trials containing semantic distractors, which were ultimately answered correctly. We conclude that the VWP is a useful tool for investigating retrieval interference effects, including both the online effects of distractors and their after-effects, when repair is initiated. This work paves the way for further studies of retrieval interference in the spoken modality, which is especially significant for examining the phenomenon in pre-reading children, non-reading adults (e.g., people with aphasia), and spoken language bilinguals.

## Introduction

Memory processes are crucial for language comprehension, especially the ability to store linguistic constituents and retrieve them later (perhaps much later) to combine with new information. For example, it is quite common for linguistically dependent information to be separated by a considerable distance. An example of such a construction is in (1), where a dependent constituent, *the girl*, is separated from the verb *smelled* by two relative clauses.

(1) The girl who walked with the cute little boy that wore the striped shirt smelled the flowers.

Consequently, a clear understanding of the memory processes that support accurate comprehension is critical to any psycholinguistic model of language use. In this paper, we present a novel application of the Visual World eye-tracking Paradigm (VWP; Altmann, 2004; Trueswell and Tanenhaus, 2005) for studying these memory retrieval processes in spoken language comprehension. The particular novelty of the current study is to test the VWP against the logic of the dual-task paradigm, which has been used previously (Van Dyke and McElree, 2006, 2011) as a means of explicitly manipulating the contents of memory, and arguing specifically for retrieval interference (as opposed to encoding interference) in processing of spoken sentences with syntactic dependencies.

### The cue-based retrieval theory (CBRT)

Several theories have been proposed to explain why establishing memory-dependent linguistic relationships as in (1) is challenging, even for monolingual adult speakers (see Levy et al., 2013 for a review). One of the most cited is *the Cue-Based Retrieval Theory* (*CBRT;* Gordon et al., 2002; McElree et al., 2003; Van Dyke and Lewis, 2003; Lewis et al., 2006; Van Dyke and McElree, 2006, 2011; Van Dyke and Johns, 2012; Van Dyke et al., 2014) which is grounded in a large body of empirical research pointing to a severely limited active memory capacity even for skilled monolingual readers, accompanied by a fast, associative retrieval mechanism that uses cues to access memory directly (reviewed in McElree, 2006). A central prediction of the CBRT is that interference effects will arise whenever retrieval cues necessary for identifying a distant dependent are ambiguous. It is this interference that creates comprehension difficulty. For example, in (1) the verb *smelled* selects for an animate subject, and there are two such NPs that fit these cues (*the girl* and *the cute little boy*). The second NP serves as a distractor for retrieving the target subject, resulting in longer reading times at *smelled* and lower accuracy to comprehension questions (Van Dyke, 2007).

In order to distinguish the retrieval account of the CBRT from accounts emphasizing costs associated with storing multiple similar items (e.g., Gordon et al., 2002). Van Dyke and McElree (2006) directly manipulated the relationship between the contents of memory and the cues available at retrieval. To do this, they utilized a dual task paradigm in which they asked participants to read written sentences like (2) in a phrase-by-phrase manner while performing a simultaneous memory load task.

(2) It was the button that the maid who returned from vacation spotted in the early morning.

On high memory load trials, participants were asked to remember a list of three words (i.e., KEY-PEN-EARRING) and then read the sentence in (2). The manipulation of interest was when the verb *spotted* was replaced with *sewed*; in the *spotted* case, all of the words from the memory list could serve as the verb's object, but only a *button* is *sew*-able. The authors observed increased interference effects from the words in the memory list in the form of longer reading times at the verb *spotted* (578 ms), but not at the verb *sewed* (540 ms). This difference disappeared when the memory list was not presented (564 vs. 567 ms, respectively), demonstrating that the reading time difference was *not* simply related to a difference in the semantic association between the verb and the clefted NP. Interference was due to the match between the distractors in the memory list and the semantic retrieval cues from the verb that specify the target referent (*the button*), i.e., an object that can be spotted.

This type of interference has now been demonstrated not only in measures of reading speed, but also in comprehension accuracy and grammaticality judgments, and in a variety of linguistic constructions; it takes place whether the intruders occur before (proactive interference) or after (retroactive interference) the retrieval target (Van Dyke and McElree, 2006, 2011; Martin and McElree, 2009); whether the intruder is syntactically, semantically, or referentially similar (Gordon et al., 2002; Van Dyke and Lewis, 2003); or even when the intruder is unlicensed in the grammatical construction (Van Dyke, 2007; Vasishth et al., 2008; Martin et al., 2012). Finally, sensitivity to interference appears to be modulated by individual differences in cognitive abilities (Van Dyke et al., 2014).

### Written vs. spoken modality

The evidence associated with the CBRT is robust, but so far, it has been restricted to the reading modality. Hence, the role of retrieval interference in *spoken language comprehension* remains unknown. Speech contains a variety of spoken cues, but there is little evidence about how spoken cues are considered by the retrieval mechanism, and what priority they may receive vis-à-vis other cues (e.g., semantic, syntactic). This issue is critical because speech cues play a primary role in memory encoding (Baddeley, 1966, 2012; Liberman et al., 1972), creating the possibility that input modality may be an important means for modulating effects of retrieval interference.

Modality effects have been found elsewhere in the literature. Using a self-paced listening paradigm, contrasted with a self-paced reading paradigm, older adults have been found to take longer to read relative clauses than to listen to them (Waters and Caplan, 2005; Caplan et al., 2011). Further, a study with cleft sentences of the sort investigated here (DeDe, 2013) examined whether input modality and syntactic complexity interact in healthy younger and older adults and people with aphasia. As in the studies conducted by Caplan and colleagues, DeDe found that the processing time for healthy controls was longer in the self-paced reading experiment than in the self-paced listening one, and this effect was only observable on the verb. She concluded that “…listening may exert fewer processing demands because it is a more natural and over-practiced skill than reading” (p. 11).

In contrast, neuroimaging studies have found small, but consistent modality differences in word (Chee et al., 1999) and sentence processing (Michael et al., 2001; Rüschemeyer et al., 2006), with listening being more resource-demanding. For example, Michael and colleagues compared subject and object relative clauses and found increased hemodynamic response in listening to object relatives in the auditory modality, but not while reading. A possible explanation for this difference, offered by Chee and colleagues, points to the greater reliance on working memory in spoken language comprehension (but see Van Dyke et al., 2014 for an alternative view). Hence, examining retrieval interference in the spoken modality and the specific role of speech cues is an important means of advancing the CBRT.

## Applying the VWP to study retrieval interference in spoken language comprehension

In all of the aforementioned studies that tested the CBRT, sentences with filler-gap dependencies were presented to participants in the written form and, therefore, effects of distractors were indirectly inferred from differences in reading times at the verb (*spotted* took longer to read than *sewed*), contrasted with similar conditions containing no extra-sentential distractors. The current study seeks to determine whether retrieval interference effects can be found in the spoken modality.

### The visual world paradigm

**Figure 1:**
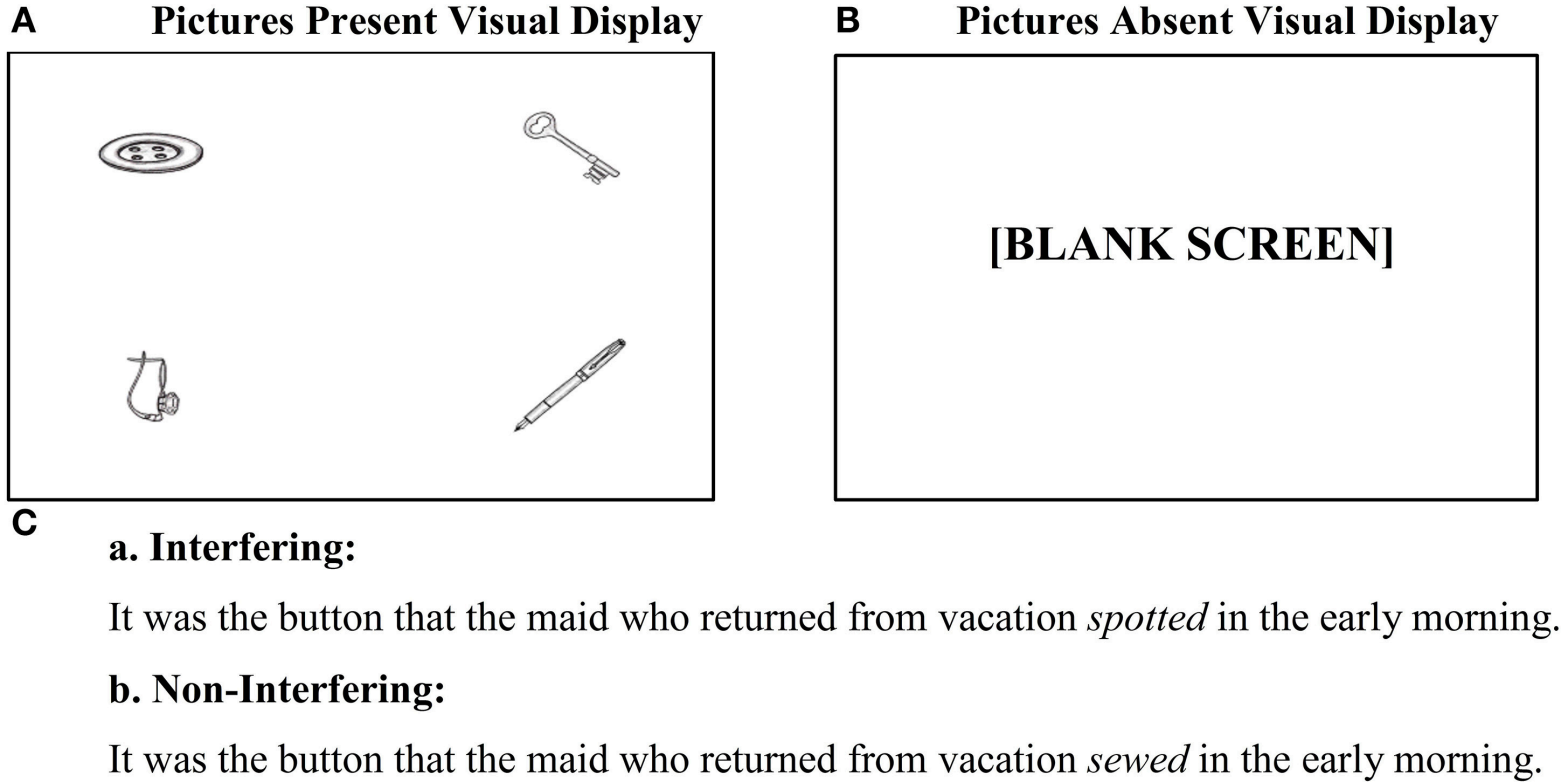
**Sample experimental conditions**. **(A)** Visual display for Picture Memory List Present, **(B)** Visual display for Picture Memory List Absent, **(C)** Two types of spoken sentences.

The Visual World eye-tracking Paradigm (VWP) is well-suited for addressing these questions because VWP experiments measure overt looking to multiple, clearly separable referents (represented as pictures or real objects) called *Target, Competitor*, and *Distractors*. Hence, it provides a straightforward measure of competition between referents while listening. For example in Figure 1, the key manipulation involves the relationship between the four pictures and the main verb in the spoken sentence. As in the original study (Van Dyke and McElree, 2006), we expect that the semantic properties of the verb will guide the search for a filler for the gapped object (trace position), so that when the verb is *sewed* there will be more looks to the button than to any other picture, whereas the verb *spotted* will support looks to any of the four pictures, which are all objects that could be spotted.

This prediction is similar to classic findings in the VWP literature, where properties of a verb enable participants to anticipate what will be referred to post-verbally (e.g., Kamide et al., 2003; Huettig and Altmann, 2005). For example, Altmann and Kamide (2004) presented participants with four pictures (i.e., a cake, toy train, toy car, and ball) while requiring them to listen to either of the two spoken sentences, (3a) or (3b):

(3) a. The boy will eat the cake.
b. The boy will move the cake.

They found that the participants were much more likely to launch eye movements to the cake in (3a) than (3b) and that this happened before the onset of the word *cake*. They interpreted these results as evidence that semantic properties of the verb are used immediately (and incrementally) to guide subsequent integrative processing.

There are several previous VWP studies that investigated processing of memory-dependent linguistic relations in sentences with syntactic dependencies. In these studies, the visual display always included four pictures of the referents explicitly named in the preamble and experimental instruction. Sussman and Sedivy (2003) tested unimpaired adults and established that in oblique object *Wh*-questions (e.g., *What did Jody squash the spider with*?), the *wh*-filler *what* triggered an increase in anticipatory fixations to the potential argument of the verb (i.e., the spider) during the verb despite the fact that the gap was filled. At the preposition, the participants quickly switched to the correct referent (i.e., the shoe). Dickey et al. (2007) simplified the object *Wh*-questions used in Sussman and Sedivy's experiment by removing the oblique object (e.g., *Who did the boy kiss that day at school?*) and compared eye movements of control adults with those of people with aphasia who had difficulties with comprehension of sentences with syntactic dependencies. Based on eye-movement patterns of people with aphasia in the incorrectly answered questions, they argued that their comprehension errors were caused by late-arising competition between the target object referent (e.g., the girl) and the competitor subject (e.g., the boy).

However, neither Sussman and Sedivy (2003) nor Dickey et al. (2007) explained their results in terms of retrieval interference. In contrast, Sheppard et al. (2015) specifically tested *the intervener hypothesis* in search for an explanation of comprehension failure in people with aphasia when they process two types of object *Wh*-questions (e.g., *Who* vs. *Which mailman did the fireman push yesterday afternoon?*). To ensure the felicity of the *which*-questions, the 4-referent display was replaced with an action picture in which one fireman and two mailmen were depicted in two simultaneous pushing events. The results suggested that the more people with aphasia looked at the incorrect mailman (i.e., the intervener) the more likely they were to answer the question, in particular, the *which*-question, incorrectly. A similar explanation was proposed by Clackson et al. (2011) in accounting for eye movements of adults and children in sentences with referentially ambiguous personal pronouns (e.g., *He [Peter] watched as Mr. Jones bought a huge box of popcorn for him*.). Children were especially prone to look more at the gender-matched referent (e.g., Mr. Jones) in the position intervening between the pronoun (e.g., *him*) and its accessible antecedent (e.g., *Peter*) even though this intervener is ruled out by the Binding theory.

Our current application of the VWP provides a more direct way of testing retrieval interference in processing of sentences with syntactic dependencies. All of the previous studies required referent selection based on a forced choice between two referents explicitly named in the spoken materials, i.e., the target and competitor. In the 4-referent set-up employed by Sussman and Sedivy (2003), Dickey et al. (2007), and Clackson et al. (2011), the remaining 2 referents (i.e., a distractor and a location) attracted very few looks, thus, effectively restricting referential choice to two. In addition to the fact that all 4 distractor referents were explicitly named in the spoken context, the intervener was placed in the sentence between the filler and gap which increased their salience and availability during retrieval of the filler at the verb.

The case study described in this article employed the dual-task paradigm (Van Dyke and McElree, 2006), in which every one of the three distractor referents was a legitimate semantic intruder that was *outside* the spoken sentence. Hence, any interference from the distractors suggests that information contained within memory, but not part of the sentence itself, impacts successful retrieval of the actual target. This has important ramifications for the specification of the type of retrieval mechanism (i.e., one that matches to all contents of memory simultaneously, as in a global matching mechanism (e.g., Clark and Gronlund, 1996) or else a retrospective serial search that entertains each item in memory individually. The former predicts that all distractors should receive increased looks when they match retrieval cues from the verb, while the latter predicts that only the target referent (which is the most recent) would receive looks from the verb. In addition, interference effects from extra-sentential distractors suggest that sentence processing utilizes the same memory capacity as that used for short-term memory, contrary to accounts that would give sentence processing a separate memory capacity (e.g., Caplan and Waters, 1999).

Using the VWP for studying retrieval interference in spoken language comprehension brings an additional advantage in that this method removes potential confounds related to reduced reading skill or difficulty comprehending complex task instructions, concerns which are paramount when investigating comprehension ability in linguistically diverse populations, such as children, bilingual and second language learners, and participants with language impairments. Instead, the VWP provides a naturalistic way to assess language processing while participants listen to verbal input and look at visual arrays. In addition, it could be employed in a passive listening mode that does not require verbal, gestural, or motor responses, making it amenable for use with older individuals or persons with aphasia (Hallowell et al., 2002; Ivanova and Hallowell, 2012).

### “The blank screen” paradigm in the VWP

The classic VWP experiments with spoken sentences found anticipatory looks toward an object when the verb precedes it (Kamide et al., 2003; Huettig and Altmann, 2005) demonstrating that the verb's selectional restrictions activate its argument structure. The latter, in its turn, drives looks to the referent that is named by the noun in post-verbal position. However, looks could be crucially dependent on the co-occurrence of linguistic input and the overt presence of the referent's picture. To counter this argument, Altmann (2004) demonstrated that the physical presence of the pictures was not necessary. Listeners still moved their eyes to the location of a previously displayed object even when the object was no longer present while they listened to the spoken sentence. This method received the name of the “blank screen” paradigm. Although the proportion of looks using this method was relatively low in absolute terms (16%; Altmann and Kamide, 2004, Figure 11.1), Altmann and Kamide interpreted these results as evidence that it is the mental representations of the objects held in memory that are activated by the verb's semantics. Therefore, eye movements in the VWP were shown to reflect the mental world, and not just visual attention in the form of iconic memory.

Because this method has particular theoretical significance in the VWP literature, we chose to implement the blank screen paradigm as a potential analog of the Memory-Load condition of Van Dyke and McElree. We hoped this would allow us to determine the extent of interference from visually presented distractors: If interference from *absent* distractors were observed, this would suggest that semantic interference from present distractors is not merely contingent on the current visual scene, but related to accessing all matching memory representations, whether currently active or not. As it turned out, firm conclusions on this point were frustrated by a methodological confound. Hence, although we present these results, our conclusions are drawn primarily from the Pictures Present conditions in our design.

In what follows below, we present a VWP implementation of the Van Dyke and McElree (2006) study, which examined how semantic properties can be used to guide retrieval of previously occurring constituents. Specifically, in (2), the grammatical encoding of the clefted NP makes it unambiguously identifiable as the object of a later occurring verb however, there is no prospective information about the semantic relationship between that object and the verb. Thus, any difference in looks to the target in the Interfering (e.g., *spotted*) vs. Non-Interfering (e.g., *sewed*) conditions has to occur only once the verb is heard (or after) and must be attributed to interference driven by the verb's semantic cues. The prediction of fewer looks to the correct target picture (*button*) in the Interfering conditions compared to the Non-Interfering conditions is analogous to the finding in Van Dyke and McElree, where semantically similar distractors *outside* the sentence produced inflated reading times at the point of integrating the verb with its direct object.

## A case study: Retrieval interference in spoken language comprehension

### Participants

Twenty-four undergraduate students from the College of Staten Island participated in this study for credit as one of the requirements for an introductory psychology class. All participants (7 men, mean age = 21.4) identified themselves as native English speakers. This study was carried out in accordance with the ethical principles of psychologists and code of conduct of the American Psychological Association and was approved by the Institutional Review Board of the College of Staten Island. All participants gave written informed consent in accordance with the Declaration of Helsinki.

### Materials

Each experimental item was realized as one of four conditions in a 2 × 2 (Interference × Picture) factorial design. The interference manipulation was identical to that in the original study by Van Dyke and McElree (2006), but the objects from the memory set were presented as pictures, and not as words: in the interfering condition, all pictured items could serve as the object of the main verb (e.g., *spotted*) in the sentence. For the corresponding non-interfering condition, the same pictures were presented but the main verb was changed in the spoken recording (e.g., *sewed*) so that only the clefted NP made sense as its object (See Figure 1). Each picture occupied one of the four quadrants on the stimuli computer monitor, and the clefted NP picture was evenly rotated through each quadrant. For the picture manipulation, pictures remained on the screen while the sentence played (Present) or were removed (Absent, the blank screen paradigm) after the participant named them.

As in Van Dyke and McElree's (2006) Memory Load conditions, the picture memory list was always presented to participants first—prior to reading the spoken sentence, as in (2). The sentence was always followed by a *yes/no* comprehension question, and then, finally, they were asked to recall the four pictures from the memory list. The four steps of the procedure were each crucial to the implementation of the memory interference paradigm. The picture memory list established potential distractors in the comprehension context, the sentence presented the main language processing task, the comprehension questions ensured that participants would attend to the sentence (rather than ignore it in favor of focusing all their attention on the memory task), and the recall task ensured that they would work to keep the pictures from the memory list within their active memory. Participants were explicitly told to do their best on each of the individual tasks.

An important dimension of exploring retrieval interference in the spoken modality is the possible effect that prosodic cues may play in mediating retrieval difficulty. It is currently not known whether or not these cues are considered by the retrieval mechanism, and what priority they may receive vis-à-vis other cues (e.g., semantic and syntactic). Because of this, we decided to employ neutral prosody so as to establish a baseline for whether the expected effects would manifest in eye-movement patterns. Although clefted constructions such as (2) often occur with a stress contour, there is no information about whether individual readers assign such a contour when they read them silently. This is significant because the original study by Van Dyke and McElree (2006) employed self-paced reading, which may have discouraged the natural assignment of implicit prosody. Thus, we considered the use of neutral prosody to be the best approximation to the reading conditions in the original study.

The 28 sets of experimental items were selected from the original 36 object cleft sentences of Van Dyke and McElree's (2006) self-paced reading experiment based on how well the items in the memory lists could be depicted. There were also 56 filler items of two types: eighteen subject cleft sentences (e.g., *It was the son who was wild that smashed the lego tower that nearly reached the ceiling*.—Picture Memory List: ROSE, POMEGRANATE, SICKLE, VIOLIN), and 38 non-clefted sentences (e.g., *The sailors knew that the treasure enticed the pirate on the hijacked ship*—Picture Memory List: HOUSE, STAR, ROBE, FAIRY). Pictures for the filler sentences were selected randomly; one half was presented with pictures, and the other half was paired with a blank screen. There were also five practice items with feedback. Four lists were constructed using the Latin Square design consisting of five practice, 28 experimental (7 items per condition) and 56 filler items in such a way that each experimental item was both preceded and followed by one of the fillers. Thus, all experimental items were separated by two fillers. Six participants were randomly assigned to each of the four lists, containing 89 trials in total.

The 356 pictures (89 trials × 4 pictures) were selected from the electronic database of object and action pictures created in the Neurolinguistics Laboratory (head: Dr. Olga V. Dragoy) at the Higher School of Economics (Moscow, Russia). The database is available online free of charge (http://stimdb.ru/) and contains black-and-white pictures normed on many dimensions (i.e., naming agreement, visual complexity, age of acquisition, frequency, and familiarity; Akinina et al., 2015).

All spoken sentences (experimental and filler) were recorded by a female native speaker of American English at a sample rate of 22,050 Hz. Every effort was made to pronounce them with neutral prosodic intonation to eliminate the contribution of special prosodic cues associated with cleft sentences in English, i.e., a fall-rise pitch accent on the clefted NP (Hedberg, 2013) and a prosodic break after the clefted NP indicating phrasal boundary, during retrieval. However, after data collection we discovered that this goal was not met: experimental sentences were recorded in two different sessions, which resulted in subtle perceptual and prosodic differences between the interfering and non-interfering conditions. We discuss this methodological error later. Speaking rate was slightly slower than is heard in everyday casual speech, due to efforts to enunciate each word; see Appendix B in Supplementary Material for example recordings.

The comprehension questions were designed following the method of Van Dyke and McElree (2006). Two thirds of the questions for the experimental items (19 out of 28) were about the subordinate clause (e.g., Example 4: *It was the cigarette that the criminal who robbed the electronics store smoked/sought in the dark alley*. Question: *Did the criminal rob a liquor store?*) and one third (9 items) were about the main clause with the clefted NP (e.g., for Example 2 the question was, *Was it the maid who was on vacation?*).

The pictures, spoken sentences, and comprehension questions for all 28 experimental items and a sample of eight representative fillers, as well as the two auditory versions of example (2) in both the interfering and non-interfering conditions, are provided in Audios 1 and 2 in the Supplementary Material.

### Procedure

The experiment was controlled by DMDX software (Forster and Forster, 2003), with the game pad serving as the interface device. Participants were seated in front of a 17-inch Dell laptop (resolution of 1024 × 768 pixels) at a viewing distance of ~60 cm. On each trial, participants first saw the four-picture memory list (Figure 1A), with each picture centered in one of the four 350 × 350-pixel quadrants of the display. Each of the four images subtended about 11 degrees of visual angle. Participants were asked to label the pictures in any order using just one word and then press the “Yes” button on the game pad to listen to the auditory sentence (Figure 1C, a-b). Specific picture labels were not sought in this experiment, hence no feedback was given in this phase. In the pictures present condition, participants continued to look at the pictures while listening to the sentence (Figure 1A); in the pictures absent conditions, they looked at the blank screen (Figure 1B). An auditory comprehension question automatically followed the sentence (e.g., *Was it the maid who was on vacation?*) and was answered by pressing either the “Yes” or “No” button on the game pad. As soon as the response was provided, DMDX presented a written reminder for the participants to recall the four pictures from the memory list (i.e., *Now recall the four pictures*), and their voice responses were recorded with the help of a microphone connected to a digital SONY DSR-30 video tape-recorder. Participants were asked to recall all of the pictures in any order, but were encouraged not to belabor the recall if they couldn't remember them.

The video tape-recorder was connected to the ISCAN ETL-500 remote eye-tracking system that collected participants' eye movements. Eye movements were sampled at a rate of 30 times per second. Prior to the experiment each participant underwent a short calibration procedure. The experiment was conducted in one session and lasted ~1 h.

### Statistical analysis

Mixed-effects logistic regression was used to examine three measures: picture recall accuracy, comprehension question accuracy, and eye movement data. Mixed-effects modeling allows us to account for the clustered nature of the data, with responses nested within participants and items; furthermore, it makes it possible to examine variability within and between participants and items and is flexible in handling missing data (Raudenbush and Bryk, 2002). All models included crossed random intercepts for participants and items (Baayen et al., 2008). Random slopes for the-within-subjects independent variables were examined but not retained in any of the analyses, either because of convergence failure or because the random slopes did not improve the model fit.

Between-subjects outliers were trimmed following a 2-stage procedure: first, for each experimental condition we excluded subjects with average proportion of fixations more than 2.5 SD below or above the grand mean. Second, for each model, we examined the level-2 residuals and we re-fitted the models without observations with absolute standardized residuals greater than 2.5. This 2-stage procedure never led to the exclusion of more than 3% of the data.

Missing values due to equipment malfunctioning and track loss constituted 0.4 and 4.6% of the data, respectively. Data were analyzed with R version 3.1.2 (R Core Team, 2014) using the glmer function from the *lme4* package, version 1.1-7 (Bates et al., 2014).

## Results and discussion

### Recall of pictures

In the beginning of the trial, participants were asked to label each picture in the 4-item memory list using one word, but they were free to choose any appropriate word. For example, they could choose to label a picture depicting a rug as “*carpet*.” No feedback or corrections were provided except in the practice trials. Accuracy of recall of pictures was scored based on the actual number of pictures recalled for each trial and ranged from zero (no pictures recalled) to 100% (all four pictures recalled). Any order of recall was allowed as long as the pictures were labeled the way the participant labeled them in the beginning of the trial (e.g., saying *rug* when the picture was labeled as *carpet* was counted as an error). The top row of Table 1 shows the mean correct recall of the pictures as a factor of Interference and Picture.

**Table 1:** **Accuracy of recall of pictures and comprehension questions, % (SD)**.

	**Interfering, Pictures Present**	**Interfering, Pictures Absent**	**Non-Interfering, Pictures Present**	**Non-Interfering, Pictures Absent**
Picture recall	91.4 (8.17)	86.3 (12.8)	91.7 (7.19)	85.3 (12.0)
Comprehension question	32.1 (20.3)	29.2 (25.8)	35.1 (17.4)	35.1 (17.4)

Mixed-effects logistic regression analysis was used to examine the effect of Interference and Picture on accuracy of picture recall. Results showed a significant effect of Picture, such that the recall of the pictures was significantly better in the Pictures Present than in the Pictures Absent conditions, 91.6 vs. 85.8% (cf. Table 2, left panel). There was no effect of Interference and no interaction. Interestingly, the recall of the pictures in our experiment was higher than that for written word memory lists in Van Dyke and McElree's (2006) experiment (non-interfering condition: 80%, interfering condition: 78% in that study) and this was true even in the Pictures Absent condition. We interpret this as evidence that visually presented items have increased salience in memory as compared to verbally encoded memory words. This could possibly be explained by the difference in encoding modality: an auditorily presented sentence interferes less with memory for visually encoded stimuli. It is also possible that recall was increased because participants had both a visual and verbal encoding of the stimuli (Nelson and Brooks, 1973; Snodgrass and McClure, 1975; Paivio, 1986).

**Table 2:** **Accuracy of recall of pictures and comprehension questions: Summary of mixed-effects logistic regression analyses (fixed effects only)**.

	**Picture recall**				**Comprehension questions**			
**Variable**	**Estimate**	**SE**	***z***	***p***	**Estimate**	**SE**	***z***	***p***
(Intercept)	2.108	0.284	7.417	<0.001	−0.775	0.265	−2.922	0.003
Interference	0.527	0.393	1.342	0.180	−0.283	0.260	−1.087	0.277
**Picture**	**0.969**	**0.424**	**2.286**	0.022^*^	0.048	0.254	0.189	0.850
Interference × Picture	−0.754	0.613	−1.230	0.219	0.018	0.366	0.050	0.960

* *p < 0.05*.

### Comprehension question accuracy

Accuracy of responses to the comprehension questions as a factor of Interference and Picture was low overall, 32.9% (see Table 1, bottom row.) Results of mixed-effects logistic regression analysis of accuracy showed no significant effects (see Table 2, right panel). This is consistent with the results in Van Dyke and McElree (2006), however despite no significant effects the participants in that study had much higher accuracy levels (87% in the Non-interfering condition vs. 83% in the Interfering condition, a statistically significant difference). We note that this low accuracy was not due to our participants' overall level of performance in the experiment—their overall high picture recall (88.7%) confirms that they did pay attention. One possible explanation for the difference between the current results and the Van Dyke and McElree results is that the latter used the self-paced reading method which allows participants to read at their own pace. This self-controlled, and likely slower, presentation rate affords participants additional time for encoding and/or deciphering the meaning of the sentence, which in turn positions them to do better on the comprehension questions. In contrast, the spoken sentence passes quickly in the listening paradigm used here, and together with memorizing the pictures, this may have made the task more difficult. This is consistent with other findings showing less accurate comprehension in the auditory modality compared with comprehension of the same sentences in the written modality (Johns et al., 2015). Another possibility, suggested by our comparatively higher recall accuracy, is that participants traded off attention during sentence reading with attention to the recall task. We discuss this further below.

### Eye movements

**Figure 2:**
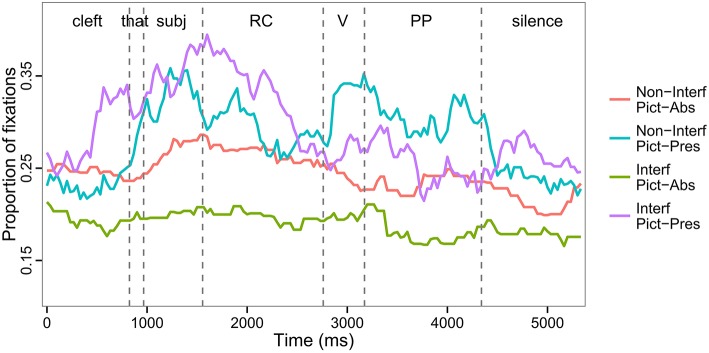
**Time course of fixation to the target picture as a function of Interference, Picture Memory List, and Region (all trials)**.

The spoken sentences were divided into four regions for purposes of statistical analysis of eye movements: three sentence regions illustrated in (4) and one second of silence following the end of the sentence. The actual durations of each ROI in individual sentences varied because of differences in lexical items that constituted the experimental items. Each ROI was constructed around the specific onsets and offsets of individual items, but in the time course figures (Figure 2), the vertical dashed lines are aligned with the average onsets of the 4 ROIs.

**Table:** 

**(4)**	**Region 1 Clefted NP-that-Subject-Relative Clause (RC)**	**Region 2 Verb**	**Region 3 PP**	**Region 4 Silence**
	It was the button that the maid who returned from vacation	sewed/spotted/	in the early morning	

Eye movements were coded from the launch of a saccade to one of the 4 referent pictures present in the visual display and included a fixation that followed, as long as their combined duration was at least 100 ms. Looks in between the referents were coded as *else*, and looks off the screen were considered track loss and were removed from statistical analysis. Descriptive statistics and a graphical representation of the time course of the proportions of fixations to the target picture over all trials and all regions are reported in Table 3 and Figure 2, respectively.

**Table 3:** **Proportions of looks to the target picture as a function of Interference, Picture, and Region, mean (SD)**.

**Condition**	**Region 1**	**Region 2: V**	**Region 3: PP**	**Region 4: silence**
Interfering, Pictures Absent	0.193 (0.131)	0.214 (0.166)	0.186 (0.130)	0.179 (0.144)
Interfering, Pictures Present	0.323 (0.118)	0.269 (0.162)	0.259 (0.115)	0.252 (0.151)
Non-Interfering, Pictures Absent	0.259 (0.127)	0.259 (0.170)	0.236 (0.132)	0.212 (0.139)
Non-Interfering, Pictures Present	0.281 (0.113)	0.294 (0.205)	0.306 (0.150)	0.243 (0.113)

#### Region 1: Clefted NP-that-subject-RC

**Figure 3:**
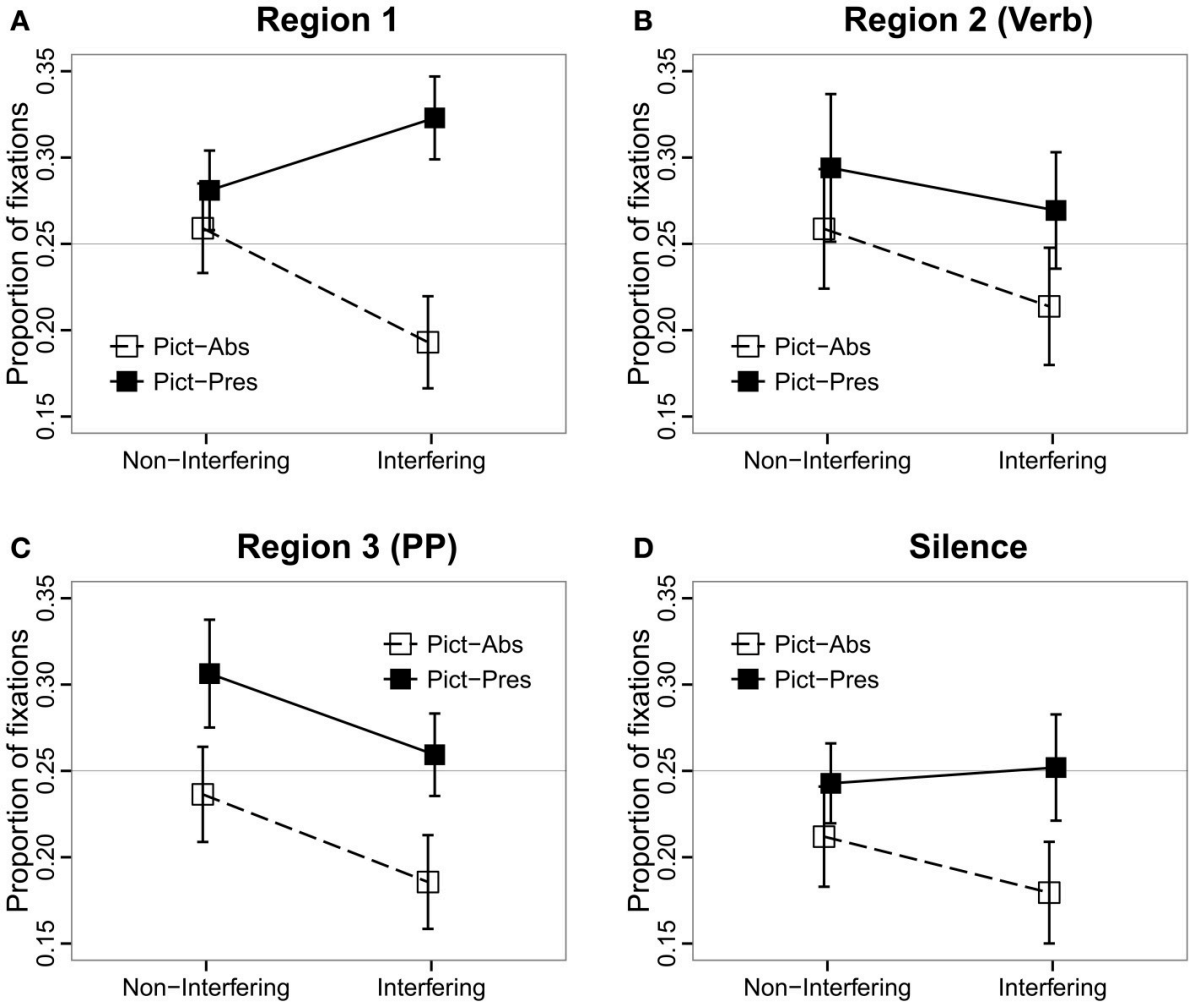
**Proportions of fixations to the target picture as a function of Interference, Picture Memory List, and Region**. **(A)** Region 1 (Clefted NP-that-Subject-RC), **(B)** Region 2 Verb, **(C)** Region 3 (PP), **(D)** Region 4 Silence.

Results of mixed-effects logistic regression analysis (Table 4; Figure 3A) showed a to-be-expected significant effect of Picture, such that the proportion of looks to the quadrant of the target picture was greater in the Pictures Present condition than in the Pictures Absent condition, where the eyes may be more apt to roam around the blank screen. Unexpectedly, we observed significant effects of Interference and an Interference × Picture interaction in this region, such that the proportion of looks to the target picture was greater in the Interfering than in the Non-Interfering condition when the pictures were present, and smaller in the Interfering than in the Non-Interfering condition when the pictures were absent. As both the linguistic and picture contexts were identical for all conditions prior to the verb, we trace this effect to the prosodic differences in the sentence recordings. *Post-hoc* analyses revealed that the average durations of the two components of the clefted NP—the cleft part (e.g., *it was the…*) and the target noun (e.g., *button*)—were consistently shorter in the Interfering than in the Non-Interfering condition, 309 ms vs. 343 ms [*t*_(26)_ = 4.5496, *p* < 0.001], and 323 ms and 346 ms, respectively [*t*_(26)_ = 4.0341, *p* < 0.001]. In addition, twice as many sentences in the Interfering condition (i.e., 15) than in the Non-Interfering condition (i.e., 8) had an extra prosodic break after the clefted NP (4) (// indicates a prosodic break). A representative pair of the actual recordings of the sentence types (e.g., Audio 1 and Audio 2) are available in the Supplementary Material.

(4) a. Interfering: It was the button // that the maid // who returned from vacation spotted…
b. Non-Interfering: It was the button // that the maid who returned from vacation sewed…

**Table 4:** **Proportions of looks to the target picture: Summary of mixed-effects logistic regression analyses by Region (fixed effects only)**.

**Region**	**Variable**	**Estimate**	**SE**	***z***	***p***
Region 1 (clefted NP+that+ Subject+RC)	(Intercept)	−1.167	0.152	−7.699	<0.001
	**Interference**	−**0.405**	**0.031**	−**12.92**	<0.001^***^
	**Picture**	**0.122**	**0.030**	**4.14**	<0.001^***^
	**Interference** × **Picture**	**0.611**	**0.042**	**14.454**	<0.001^***^
Region 2 (Verb)	(Intercept)	−1.148	0.138	−8.299	<0.001
	**Interference**	−**0.250**	**0.078**	−**3.206**	0.001^***^
	**Picture**	**0.263**	**0.078**	**3.391**	<0.001^***^
	Interference × Picture	0.072	0.109	0.659	0.51
Region 3 (PP)	(Intercept)	−1.310	0.153	−8.54	<0.001
	**Interference**	−**0.315**	**0.049**	−**6.359**	<0.001^***^
	**Picture**	**0.384**	**0.046**	**8.391**	<0.001^***^
	Interference × Picture	0.008	0.067	0.114	0.909
Silence	(Intercept)	−1.457	0.172	−8.465	<0.001
	**Interference**	−**0.229**	**0.054**	−**4.211**	<0.001^***^
	**Picture**	**0.183**	**0.051**	**3.614**	<0.001^***^
	**Interference** × **Picture**	**0.260**	**0.073**	**3.56**	<0.001^***^

*** *p < 0.001*.

We speculate that despite the fact that we avoided pitch contours in an effort to keep prosody neutral, these differences created unintended prosodic cues that served to direct looks to the target noun in the Interfering, Pictures Present condition. The fewer looks to the target in the Interfering, Pictures Absent condition may also have resulted from the increased saliency of the target item, so that looking to the now-empty location of the target referent was not as necessary as it was for remembering the other, less salient referents^1^. Whether or not this account is correct, we emphasize that with respect to the Pictures Present condition, whatever bias drove these results went in the *opposite* direction to that predicted for the critical region (Region 2), where we expected looks to the target to *decrease* in the Interfering condition as compared to looks to competitors, which should increase in response to retrieval interference. Moreover, we conducted additional *post-hoc* analyses of the region after the target noun and found no additional prosodic differences between conditions. Hence, we are confident that results in Regions 2-3 are interpretable despite this methodological error. As for the Pictures Absent condition, this is a true confound. In order to better assess the presence of the Interference effect in relation to the Picture manipulation, we report the results of pair-wise comparisons for all future analyses.

#### Regions 2–3: verb-PP

These two regions—Region 2 (Verb) and Region 3 (PP)—revealed the predicted pattern of results for the Interference manipulation. Region 2 is the critical region containing the verb that determines whether the pictured items are distractors or not (Figure 3B). We found a significant main effect of Interference, such that there were fewer looks to the target in the Interfering condition, where all of the pictured items could serve as the object of the main verb (e.g., they are all fixable in the example in Figure 1). We performed pair-wise comparisons to make sure that the main effect of Interference was not driven by the Pictures Absent condition. We found that the effect was significant in both the Pictures Present conditions (Tukey test: *z* = −2.34, *p* < 0.05) and in the Pictures Absent conditions (Tukey test: *z* = −12.93, *p* < 0.001). We also observed a significant effect of Picture, with a greater proportion of looks to the target picture in the Pictures Present condition as compared to the Pictures Absent condition. The interaction was not significant.

In Region 3, which contained the prepositional phrase, (Figure 3C) we observed the same pattern of results as in Region 2 (see Table 4). The proportion of looks to the target was greater in the Non-Interfering condition than in the Interfering condition. This effect obtained in the pairwise comparisons in both the Pictures Present conditions (Tukey test: *z* = −6.88, *p* < 0.001) and in the Pictures Absent conditions (Tukey test: *z* = −6.36, *p* < 0.001). Inspection of eye-movements in this time window (see Figure 2) suggests that this result was driven by looks to the target at the end of the sentence, and may reflect end-of-sentence wrap-up effects in which the participant is verifying his/her interpretation of the subject-verb dependency. As in the previous region, a significant effect of Picture was also observed, with more looks to the target in the Pictures Present condition.

#### Region 4: Silence

In the 1-s interval of silence following the end of the sentence (Figure 3D) the effect of Interference interacted with Picture, such that the proportion of looks to the target picture was comparable in the Interfering and Non-Interfering conditions when the pictures were present (Tukey test: *z* = 0.63, *p* = 0.78), and smaller in the Interfering than in the Non-Interfering condition when the pictures were absent (Tukey test: *z* = −4.2, *p* < 0.001). Visual inspection of these effects (Figure 2) suggests that the absence of an Interference effect in the Pictures condition, as compared to the significant Interference effect detected in the previous sentence regions, could be attributed to a proportional increase in looks to the target picture toward the end of the sentence for the Interfering conditions. We suggest that this effect can be associated with a repair process invoked when listeners realize they have constructed an incorrect interpretation due to interference from distractors. Similar late effects of semantic interference vis-à-vis retrieval cues have been observed in reading times (Van Dyke, 2007) and in BOLD signal during fMRI (Glaser et al., 2013).

#### Correct vs. incorrect trials

**Figure 4:**
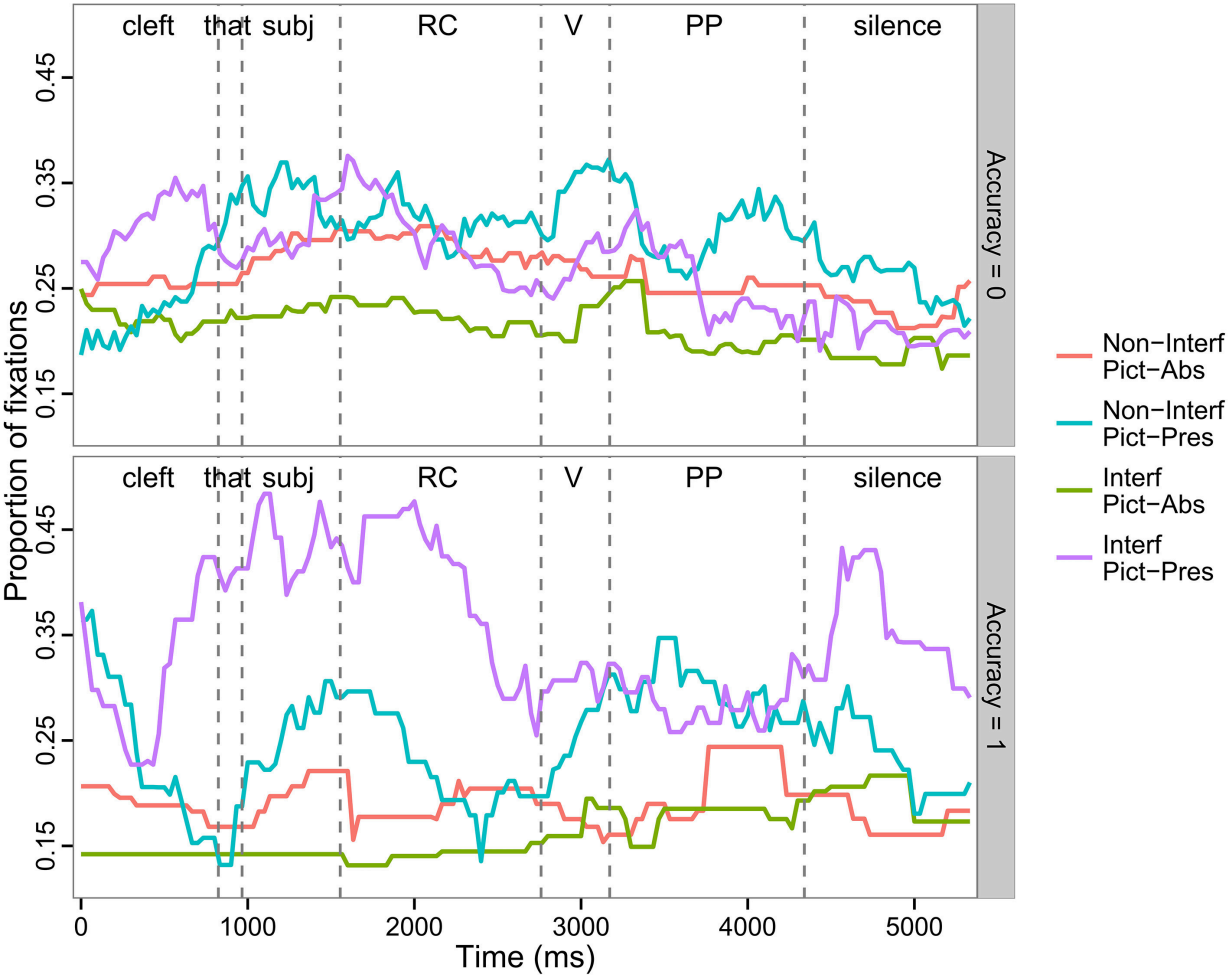
**Time course of fixation to the target picture as a function of Interference, Picture Memory List, and Region, separated by accuracy on comprehension questions**. Top panel: Incorrectly answered trials (Acc = 0), Bottom panel: Correctly answered trials (Acc = 1).

We performed a secondary analysis in which we separated the trials for which the comprehension questions were answered correctly from the ones with the incorrectly answered comprehension questions to assess the role of low accuracy on our results. Figure 4 presents the time course of fixations for both subsets of trials; Table 5 presents results of mixed-effect modeling. We observed a total of 219 correct trials, resulting in 33,356 total fixations; there was an average of 2.3 items per condition for each participant. We observed a total of 447 inaccurate trials, with a total of 69,374 fixations and 4.7 items per condition per participant. Inspection of the pattern of eye-movements in the two item subsets reveals two important observations (see Table 5 for modeling results). First, the effect of the bias toward the target in Region 1, which was created by the unintentional prosodic cues in the Interference trials, was more pronounced in accurate trials. This is apparent from the larger beta estimates in accurate trials vs. inaccurate trials (see Table 5 for main effect estimates). *Post-hoc* contrasts of the effect in the Pictures Present condition revealed a larger effect when pictures were present in accurate trials (Tukey test: β = 0.45, *z* = 9.00, *p* < 0.001) vs. inaccurate trials (Tukey test: β = 0.12, *z* = 3.62, *p* < 0.005). In particular, in the Non-Interfering, Pictures Present condition, there were more looks to the target in inaccurate (*M* = 0.29; *SD* = 0.13) than accurate trials (*M* = 0.23; *SD* = 0.17) in all 4 ROIs. As discussed in the analysis of overall results, the direction of the effect was reversed in the Pictures Absent condition, but the magnitude of beta was still larger in the accurate trials (Tukey test: β = −0.35, *z* = −5.78, *p* < 0.001) than for inaccurate trials (β = −0.29, *z* = −7.72, *p* < 0.001). This is consistent with the idea that prosodic cues in the Interfering condition served to distinguish the target, which enabled participants to more accurately comprehend the sentences. However, given that only 33% of trials were correctly answered, it appears that these prosodic cues were often not helpful for participants.

**Table 5:** **Proportions of looks to the target picture: Summary of mixed-effects logistic regression analyses by question accuracy (fixed effects only)**.

**Region**	**Variable**	**Estimate**	**SE**	***z***	***P***
**INCORRECT TRIALS**					
Region 1 (clefted NP+that+ Subject+RC)	(Intercept)	−1.071	.0639	−16.742	<0.001
	**Interference**	−**0.289**	**0.038**	−**7.714**	<0.001^***^
	**Picture**	**0.096**	**0.036**	**2.671**	<0.01^**^
	**Interference** × **Picture**	**0.414**	**0.051**	**8.161**	<0.001^***^
Region 2 (Verb)	(Intercept)	−0.947	0.137	−6.928	<0.001
	**Interference**	−**0.424**	**0.093**	−**4.579**	0.001^***^
	Picture	0.046	0.093	0.495	0.621
	Interference × Picture	0.213	0.130	1.642	0.101
Region 3 (PP)	(Intercept)	−1.091	0.094	−11.64	<0.001
	**Interference**	−**0.202**	**0.057**	−**3.583**	<0.001^***^
	**Picture**	**0.280**	**0.054**	**5.207**	<0.001^***^
	**Interference** × **Picture**	−**0.181**	**0.077**	−**2.342**	<**0.05** ^*^
Silence	(Intercept)	−1.242	0.104	−11.941	<0.001
	**Interference**	−**0.215**	**0.064**	−**3.368**	<0.001^***^
	Picture	−0.005	0.061	−0.075	0.940
	Interference × Picture	0.152	0.088	1.739	0.082
**CORRECT TRIALS**					
Region 1 (clefted NP+that+ Subject+RC)	(Intercept)	−1.458	0.163	−8.941	<0.001
	**Interference**	−**0.353**	**0.061**	−**5.782**	<0.001^***^
	**Picture**	**0.291**	**0.054**	**5.447**	<0.001^**^
	**Interference** × **Picture**	**0.806**	**0.078**	**10.341**	<0.001^***^
Region 2 (Verb)	(Intercept)	−1.592	0.245	−6.495	<0.001
	Interference	0.099	0.156	0.637	0.524
	**Picture**	**0.414**	**0.150**	**2.769**	<0.01^**^
	Interference × Picture	−0.121	0.207	−0.581	0.561
Region 3 (PP)	(Intercept)	−1.594	0.217	−7.361	<0.001
	**Interference**	−**0.658**	**0.106**	−**6.218**	<0.001^***^
	**Picture**	**0.552**	**0.088**	**6.271**	<0.001^***^
	**Interference** × **Picture**	**0.525**	**0.134**	**3.926**	<0.001^***^
Silence	(Intercept)	−2.171	0.271	−8.013	<0.001
	**Interference**	**0.397**	**0.110**	**3.597**	<0.001^***^
	**Picture**	**0.740**	**0.096**	**7.695**	<0.001^***^
	Interference × Picture	−0.002	0.137	−0.021	0.983

* *p < 0.05*,

** *p < 0.01*,

*** *p < 0.001*.

Secondly, and more importantly, the data suggest an interference effect regardless of trial accuracy, but with different time-course manifestations. For incorrectly answered trials (top panel, Figure 4), looks to the target in the Interfering condition are reduced compared to the Non-Interfering condition beginning at the critical Region 2 (Verb), and continuing on, until the end of the sentence. This main effect was significant in all regions (see Table 5); pairwise comparisons verify the finding for both Pictures Present contrasts (Region 2, Tukey test: *z* = −2.29, *p* < 0.05; Region 3, Tukey test: *z* = −0.20, *p* < 0.001; Region 4, Tukey-test: *z* = −3.04, *p* < 0.005) and Pictures Absent contrasts (Region 2, Tukey test: *z* = −4.58, *p* < 0.001; Region 3, Tukey test: *z* = −3.59, *p* < 0.001; Region 4, Tukey test: *z* = −7.29, *p* < 0.001. In contrast, for the correctly answered trials, the Interference effect seems not to arise until the later Region 3 (PP), where we observed more looks to the target in the Non-Interfering condition for both the Pictures Present and Pictures Absent conditions^2^. The reason for this later time-course seems likely related to the bias in the Interfering conditions created by prosodic cues, which encouraged more looks to the target just prior to the critical verb. For Pictures Present trials, Figure 4 shows an immediate increase in looks to the target in Region 2 for the Non-Interfering conditions, however given the already inflated baseline for looks in the Interfering condition, the difference between the two took longer to manifest. It is especially notable that even with the bias toward looks to the target in the Interfering condition, a reduction in looks to the target in that condition compared to looks in the Non-Interfering condition was anyway observed. Moreover, in the Pictures Absent conditions, a substantial increase in looks to the target in the Non-Interfering condition compared both to the previous baseline for that condition as well as the Interfering, Pictures Absent condition is also apparent. This effect did reach statistical significance (Tukey test: *z* = −6.22, *p* < 0.001). Although these data patterns are not all confirmed statistically, they are consistent with the expected effect of the non-interfering verb as providing unambiguous cues for identifying the correct filler for the post-verbal gap.

Also of note in the accurate conditions, we observed a strong “correction” to the Interference effect in Region 4 (Silence), characterized by a steep increase in looks to the target in the Interference conditions. This effect was significant for both the Pictures Present contrast (Tukey test: *z* = 4.47, *p* < 0.001) and the Pictures Absent contrast (Tukey test: *z* = 3.60, *p* < 0.001). This is the same effect referred to in the overall analysis as a “wrap-up” or repair process. We conclude that this secondary analysis supports the repair interpretation of the Region 4 effect discussed above, as it was only the correctly answered trials that drove that late effect.

## General discussion

The goal of the present experiment was to test whether the Visual World eye-tracking Paradigm can be extended to study retrieval interference in spoken language comprehension. We sought to determine whether the VWP could enable direct observation of online interference effects, through measuring overt looks to pictures of distractor referents held in memory, rather than needing to infer interference effects from reading times. The current study provides initial evidence—despite a methodological flaw—that indeed, retrieval interference effects do occur in the spoken modality, and the VWP provides a robust means of examining them. The key finding is of increased looks to extra-sentential competitors in the interference condition, which produced a concomitant *decrease* in looks to the target in this condition. This is consistent with the suggestion of Van Dyke and colleagues, that a cue-driven retrieval mechanism uses cues to query *all* of the contents of memory^3^. When the semantic cues from the verb also match the competitors, as in the Interference conditions, then this type of global matching will cause the competitors to affect processing (either by increasing reading times or engendering more looks to themselves), even though they are not in the sentence, or strongly related to each other or any other words in the sentence. The benefit of VWP paradigm is that we can directly observe the looks to the extra-sentential competitors, whereas in the original reading time studies a “No-Load” contrast condition was necessary to support the inference that the increased reading time at the verb in the interference condition was not due to a more difficult integration between the clefted NP and the verb. In what follows, we discuss our results further in relation to the original Van Dyke and McElree (2006) study.

Despite modality and methodological differences, the two studies are consistent in demonstrating effects of extra-sentential distractors on processes of argument integration. Although the dependent measures were different, i.e., eye-movement patterns over pictures vs. reading times in self-paced reading, the locus of the effect was the same across paradigms—at and after the manipulated verb, which provided either discriminating or ambiguous retrieval cues for identifying the target direct object. In the current experiment, participants looked significantly less to the target picture in the Interfering conditions than in the Non-Interfering conditions beginning at the critical verb, while in the written modality they read this verb more slowly. In both cases, we hypothesize that these effects are due to the presence of the distracting referents, be they pictures or words, which matched the retrieval cues of the verb (e.g., *spotted*) in the Interfering condition, but not in the Non-Interfering condition (e.g., *sewed*).

Moreover, the VWP proved sensitive to dynamic processes associated with recovering from incorrect retrievals, as evidenced by the marked increase in looks to the target for Interfering conditions in the silence region for correct trials. This is similar to the sentence-final effect of semantic interference from distractors *within* a sentence observed by Van Dyke (2007), however the VWP has the added benefit of providing direct evidence that the increased reading times are associated with additional processing of the target in the Interfering conditions but not in the Non-Interfering conditions. In both cases, we take this increased late effort to reflect repair processes, invoked when listeners realize they have constructed an incorrect interpretation.

Despite the weakness in the current study related to unintended prosodic cues, which may have created increased encoding opportunities for the target in the Interfering condition, the interference effect was clearly observed in the Pictures Present conditions. It attests to the robustness of both the VWP method for indexing integrative processes (e.g., Tanenhaus et al., 1995; Huettig et al., 2011) and the retrieval interference effect itself. One might have expected that the more salient target would have promoted correct integration of the clefted NP, however the eye-movement patterns suggest interference effects in both correct and incorrect trials (although low power yielded non-significant results in the latter category). This demonstrates that salience alone is not sufficient to override the immediate effects of ambiguous retrieval cues on argument integration.

We attempted to further validate this conclusion using the blank screen paradigm, where we expected the same pattern of results as in the pictures present condition. This would have replicated the Altmann and Kamide (1999) results and leant further support to the hypothesis that the looks to the target in the pictures present condition are not a mere epiphenomenon due to visual cues, but instead reflect integrative processing driven by a cue based retrieval mechanism. Unfortunately, as described above, results from the pictures absent condition were difficult to interpret. Nevertheless, there remains a significant body of research that has established that looks to target objects during sentence processing cannot be entirely attributed to visual cues, but instead reflect activation of mental representations at least partially guided by the parser (Spivey and Geng, 2001; Altmann, 2004; Altmann and Kamide, 2004; Johansson et al., 2006). Moreover, the current study replicates Van Dyke and McElree (2006) which used the exact same sentences to demonstrate interference effects in relation to retrieval of previously stored distractors. Based on these considerations we are confident in concluding that our findings in the pictures present condition reflect the memory retrieval mechanisms at work during sentence comprehension. However, we do acknowledge the need for future work to demonstrate the validity of this approach to examining interference effects in the spoken modality more generally.

A further unexpected outcome was the extremely low accuracy to comprehension questions. We believe the primary reason for low accuracy is that the dual process task is quite difficult. High scores in the picture recall suggests that participants traded off attention to that task, for attention to the sentence task, which impacted their ability to correctly answer questions. It is highly possible that answering offline comprehension questions, which require a meta-analysis of what was heard, may be difficult for these participants for reasons that are entirely unrelated to our manipulation (e.g., poor meta-analysis skills or difficulty querying the situation model). In addition, the dissociation between accuracy scores for picture recall (high) and comprehension questions (low), together with the significant effects of Interference observed in the Pictures Present conditions, suggests that performance on comprehension questions is a poor index of whether participants experienced online effects of interference. Even when the eye movement record shows evidence of interference effects, there is no guarantee that participants were able to *accurately resolve* the interference, leading to correct performance on the comprehension questions. Thus, we take the accuracy scores to be orthogonal to the main conclusion to be drawn from these data; namely, that the VWP can reliably index retrieval interference effects during spoken language comprehension. We interpret our observation of these effects in eye movements, despite low comprehension, as an even stronger indicator that the VWP is a sensitive method for these effects.

Finally, we note an additional contribution of the current study, which is to further the goal of determining which cues guide retrieval and how they are combined (Van Dyke and McElree, 2011). This study provides an initial indication that retrieval interference effects occur independently of prosodic cues. This will be an important area for future research, some of which is already occurring in our laboratories. This paper demonstrates that the VWP is a useful method for investigating these effects. In addition, the sensitivity of VWP to indexing effects of retrieval interference opens up new possibilities for evaluating predictions of the Cue-Based Retrieval Theory in non-reading populations, such as people with aphasia, children, and auditory second language learners.

## Author contributions

IS: Design, data collection, data coding; LC: Statistical analysis; JV: Design, materials, theory development; IS, LC, and JV equally contributed to writing of the article.

### Conflict of interest statement

The authors declare that the research was conducted in the absence of any commercial or financial relationships that could be construed as a potential conflict of interest.

## Footnotes

^1^An alternative account is that the increased salience may reduce the need to look back during retrieval, perhaps because the target was already in a state of increased activation. Existing eye-movement evidence is not consistent with this interpretation however, as pre-activation of a target (greater looks to the target before hearing it) has not been related to reduced looks to the target upon hearing it or later (Altmann and Kamide, 1999; Coco et al., 2015). For example, Kukona et al. (2014) manipulated initial activation through making the target more or less predictable based on its relation to the verb. In the High Predictable condition (“eat cake”) looks to the target were never less than looks to the target in the Low Predicable conditions (“move cake”).

^2^Pairwise contrasts for accurate trials are inconclusive due to the low number of observations and corresponding high variability per condition. We discuss here only the apparent pattern of looks displayed in Figure 4. When pairwise effects do reach significance, they are noted in the text.

^3^We note that any effect of extra-sentential distractors is contrary to accounts that would give sentence processing a separate memory capacity (e.g., Caplan and Waters, 1999).
