# Supplementary material for: Using the Visual World Paradigm to Study Retrieval Interference in Spoken Language Comprehension
Source: Front Psychol. 2016 Jun 14;7:873. doi: 10.3389/fpsyg.2016.00873 (PMC4905981; doi:10.3389/fpsyg.2016.00873)
Supplement: Supplementary file 1 [file Presentation1.PDF]

APPENDIX A

Experimental Materials

Item #	Pictures	Spoken Sentence: 1st verb: Non-Interfering, 2nd verb: Interfering<br>Comprehension Question
1.	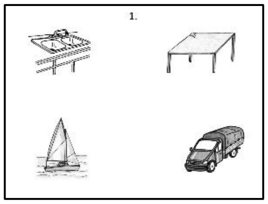	It was the boat that the guy who lived by the sea SAILED/FIXED in 2 sunny days.<br><br>Did the guy live by the sea?
2.	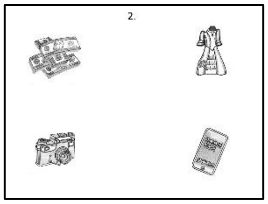	It was the dress that the waitress who worked at the diner WORE/WON the Thursday night.<br><br>Did the waitress wear the dress on Wednesday?
3.	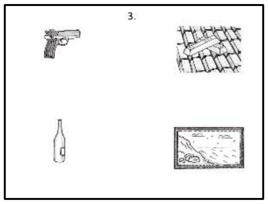	It was the gun that the character who began the fight SHOT/SEIZED near the frightened bartender.<br><br>Did the bartender start the fight?
4.	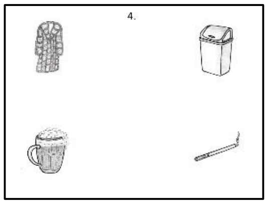	It was the cigarette that the criminal who robbed the electronics store SMOKED/SOUGHT in the dark alley.<br><br>Did the criminal rob a liquor store?
5.	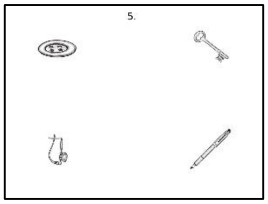	It was the button that the maid who returned from vacation SEWED/SPOTTED in the early morning.<br><br>Was it the maid who was on vacation?
6.	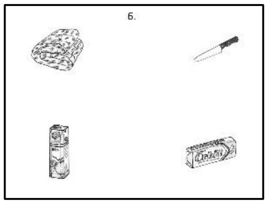	It was the gum that the camper who forgot to bring sandwiches CHEWED/FETCHED during the campfire.<br><br>Were all the campers eating sandwiches?
7.	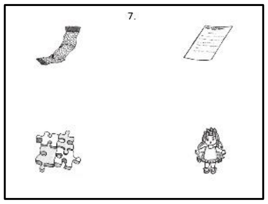	It was the puzzle that the boy who received good grades SOLVED/MISLAID after the mediocre lunch.<br><br>Was the boy doing well in school?
8.	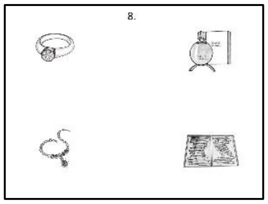	It was the perfume that the teenager who shopped for presents SMELLED/STOLE in the fancy store.<br><br>Was the perfume expensive?

Item #	Pictures	Spoken Sentence: 1st verb: Non-Interfering, 2nd verb: Interfering<br>Comprehension Question
9.	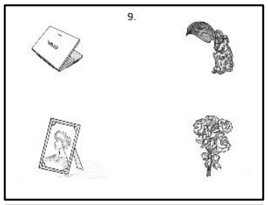	It was the hair that the student who rented the limo SHAMPOOED/DAMAGED before her prom night.<br><br>Did the student rent a limo?
10.	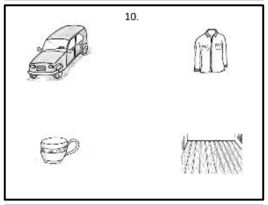	It was the car that the husband who changed his occupation DROVE/WASHED after the delicious dinner.<br><br>Was it the wife who changed her occupation?
11.	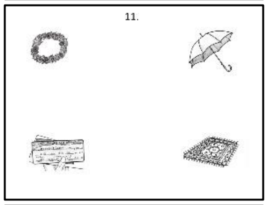	It was the rug that the house attendant who quit his previous job VACUUMED/DESTROYED on the rainy Wednesday.<br><br>Was it rainy when the rug was destroyed?
12.	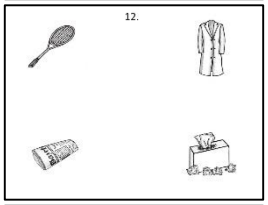	It was the newspaper that the assistant who stayed after hours PRINTED/REMOVED from the main headquarters.<br><br>Did the assistant leave work early?
13.	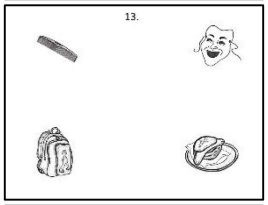	It was the hotdog that the son who entered middle school COOKED/DROPPED at the family picnic.<br><br>Did the son finish elementary school?
14.	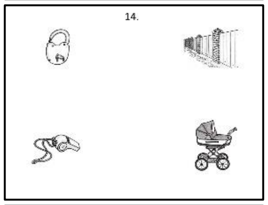	It was the whistle that the boy who listened to his mother BLEW/DAMAGED during the baseball game.<br><br>Was the boy misbehaving during the game?
15.	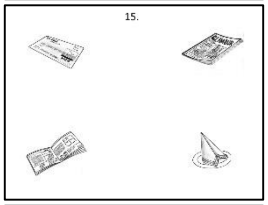	It was the card that the tourist who obtained the discount ACTIVATED/DISCARDED at the movie theater.<br><br>Did the tourist pay full price?
16.	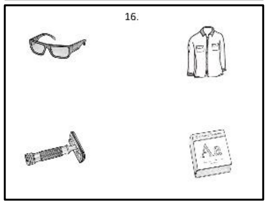	It was the jacket that the steward who traveled the world UNZIPPED/PACKED prior to the trip.<br><br>Did the steward pack the jacket?

Item #	Pictures	Spoken Sentence: 1st verb: Non-Interfering, 2nd verb: Interfering<br>Comprehension Question
17.	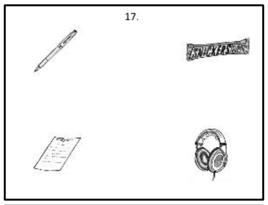	It was the report that the writer who created the show TYPED/MISPLACED after the afternoon broadcast.<br><br>Did the writer type the report?
18.	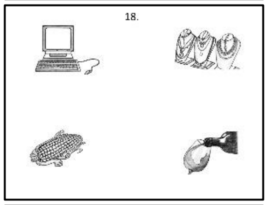	It was the computer that the associate who mastered in computer science PROGRAMMED/EXPORTED at the large company.<br><br>Was the associate an expert in business administration?
19.	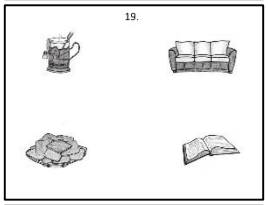	It was the book that the lady who attended church last Sunday READ/SOLD after the business meeting.<br><br>Was it the lady who was absent from the church last Sunday?
20.	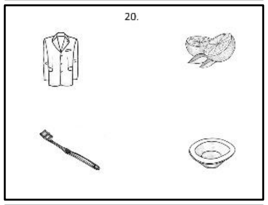	It was the orange that the traveler who lost his luggage PEELED/RINSED after the delayed departure.<br><br>Was the traveler's plane on time?
21.	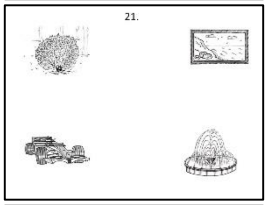	It was the bush that the wife who purchased the new house PLANTED/ADMIRED after the torrential rain.<br><br>Was the new house purchased by a man?
22.	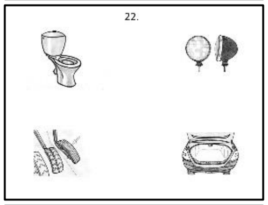	It was the pedal that the driver who worked late at night PRESSED/REPAIRED before the inspection.<br><br>Was the driver working after sundown?
23.	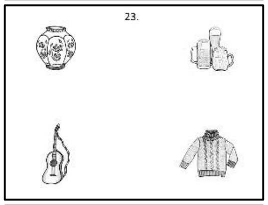	It was the alcohol that the girl who won the sweepstakes POURED/PURCHASED before the rock concert.<br><br>Was it a girl who won the sweepstakes?
24.	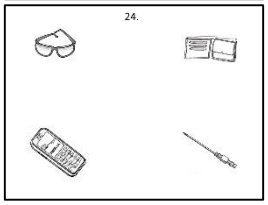	It was the needle that the physician who graduated last month INJECTED/MISPLACED before the next patient.<br><br>Was the physician very experienced?

Item #	Pictures	Spoken Sentence: 1st verb: Non-Interfering, 2nd verb: Interfering<br>Comprehension Question
25.	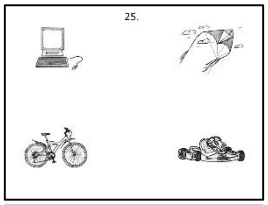	It was the kite that the youth who turned twelve years old FLEW/CRASHED after his birthday party.<br><br>Did the kite fly during the birthday party?
26.	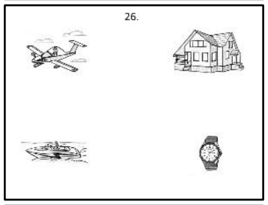	It was the plane that the brother who moved to Arizona FLEW/OWNED until the unfortunate accident.<br><br>Did the brother live in Arizona?
27.	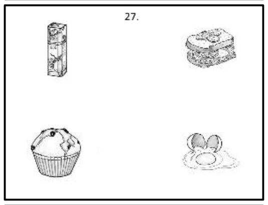	It was the egg that the policeman who received a bonus CRACKED/ORDERED in the shabby diner.<br><br>Was the policeman eating in a diner?
28.	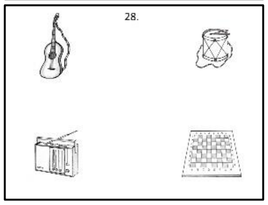	It was the radio that the adolescent who finished his homework PLAYED/UNPLUGGED in the upstairs room.<br><br>Did the adolescent play the radio?

APPENDIX B

A Representative Sample Set of Filler Items

Item #	Pictures	Spoken Sentences: #100 are subject clefts; #200 are without clefts<br>Comprehension Question
104.	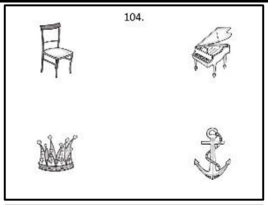	It was the fireman who was a rookie that fought the hazardous inferno that the teenager ignited.<br><br>Was the fire started by a teenager?
113.	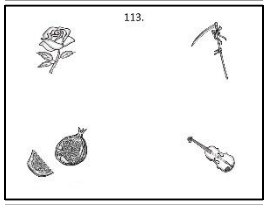	It was the son who was wild that smashed the lego tower that nearly reached the ceiling.<br><br>Was it a short lego tower?
115.	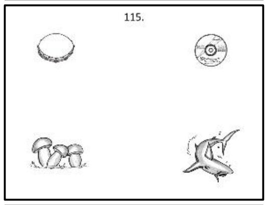	It was the girl who was exhausted that fled the violent tornado that the weatherman predicted.<br><br>Was the tornado unexpected?
116.	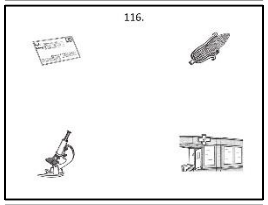	It was the daughter who was sleepy that ignored the alarm clock that rang for five minutes.<br><br>Was the daughter sleeping late?
201.	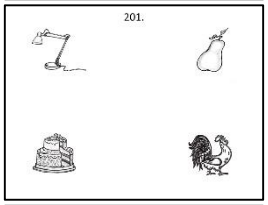	The executive knew that the clerk resented the salary that was too little to live on.<br><br>Was the salary too low?
203.	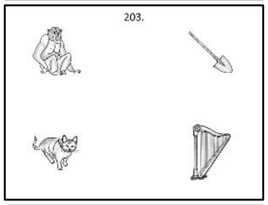	The writer remarked that the speech enraged the politician at the convention.<br><br>Was the politician enraged?
205.	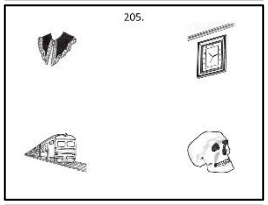	The barkeeper noticed that the bottle fascinated the alcoholic who stumbled into the bar.<br><br>Was it the alcoholic who stumbled?
208.	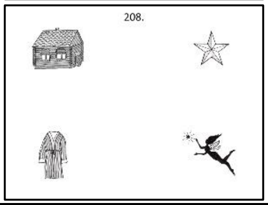	The sailors knew that the treasure enticed the pirate on the hijacked ship.<br><br>Was the pirate enticed?
